# Stabilized Perovskite Quantum Dot Solids via Nonpolar Solvent Dispersible Covalent Ligands

**DOI:** 10.1002/advs.202301793

**Published:** 2023-06-04

**Authors:** Sanghun Han, Gayoung Seo, Taeyeong Yong, Seongmin Choi, Younghoon Kim, Jongmin Choi

**Affiliations:** ^1^ Department of Energy Science and Engineering Daegu Gyeongbuk Institute of Science and Technology (DGIST) Daegu 42988 Republic of Korea; ^2^ Department of Chemistry Kookmin University Seoul 02707 Republic of Korea

**Keywords:** covalent ligands, CsPbI_3_ perovskite quantum dots, nonpolar solvents, photovoltaic absorbers, solar cells

## Abstract

The ligand exchange procedure of CsPbI_3_ perovskite quantum dots (PQDs) enables the fabrication of thick and conductive PQD solids that act as a photovoltaic absorber for solution‐processed thin‐film solar cells. However, the ligand‐exchanged CsPbI_3_ PQD solids suffer from deterioration in photovoltaic performance and ambient stability due to the surface traps, such as uncoordinated Pb^2+^ sites on the PQD surface, which are generated after the conventional ligand exchange process using ionic short‐chain ligands dissolved in polar solvents. Herein, a facile surface stabilization is demonstrated that can simultaneously improve the photovoltaic performance and ambient stability of CsPbI_3_ PQD photovoltaic absorber using covalent short‐chain triphenylphosphine oxide (TPPO) ligands dissolved in a nonpolar solvent. It is found that the TPPO ligand can be covalently bound to uncoordinated Pb^2+^ sites and the nonpolar solvent octane can completely preserve the PQD surface components. Owing to their synergetic effects, the CsPbI_3_ PQD photovoltaic absorber stabilized using the TPPO ligand solution dissolved in octane exhibit higher optoelectrical properties and ambient stability than the control absorber. Consequently, CsPbI_3_ PQD solar cells composed of PQD photovoltaic absorbers fabricated via surface stabilization strategy provide an improved power conversion efficiency of 15.4% and an enhanced device stability.

## Graphical abstract

Triphenylphosphine oxide (TPPO) ligand solution dissolved in octane is treated on ligand‐exchanged CsPbI_3_ perovskite quantum dot (PQD) solids for surface stabilization of PQDs. Through Lewis‐base interactions, the TPPO ligand exhibits strong coordination with PQD surface traps. Moreover, the nonpolar octane enables nondestructive PQD surface. Consequently, the PQD solar cells with TPPO ligand solution show improved performance and durability.

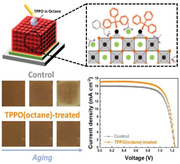

## 1 Introduction

CsPbX_3_ (X = I, Br, and Cl) perovskite quantum dots (PQDs) have received a significant attention in various optoelectronic applications, such as light‐emitting diodes, photodetectors, and solar cells, because of their high photoluminescence (PL) quantum yield, defect tolerance, and size‐ and composition‐tunable optical bandgaps.^[^ ^1^, ^2^, ^3^, ^4^, ^5^ ^]^ In particular, CsPbI_3_ PQDs have been widely adopted as a photovoltaic absorber for high‐efficiency and next‐generation thin‐film solar cells owing to their various advantages such as an appropriate optical bandgap for single‐junction solar cells and high cubic‐phase stability induced by large lattice strain.^[^ ^2^, ^6^, ^7^, ^8^ ^]^ Moreover, CsPbI_3_ PQDs pre‐crystallized via colloidal synthetic routes are prepared in a colloid ink form and thus enable the fabrication of PQD thin‐films without thermal annealing, allowing a room‐temperature process for mass production.^[^ ^9^, ^10^, ^11^ ^]^ Since the first power conversion efficiency (*PCE*) of 10.77% was reported in 2016,^[^ ^2^ ^]^ the *PCE* of CsPbI_3_ PQD solar cells has been improved up to a 16.6% through the advances in PQD material designs and device architecture engineering.^[^ ^12^, ^13^, ^14^ ^]^

High‐quality and monodispersed CsPbI_3_ PQDs are synthesized using long alkyl chain ligands such as oleic acid (OA) and oleylamine (OLA).^[^ ^15^, ^16^ ^]^ Although long‐chain ligands are necessary for the preparation of high‐quality PQDs, their insulating properties hinder the inter‐dot charge transport ability.^[^ ^17^, ^18^ ^]^ Therefore, a ligand exchange procedure to replace long‐chain ligands with short‐chain ligands is required to improve the charge transport ability for the fabrication of conductive CsPbI_3_ PQD solids. In general, a ligand exchange procedure has been performed in two steps to exchange the respective OA and OLA ligands to the ionic short‐chain ligands dissolved in polar solvents.^[^ ^8^, ^19^ ^]^ First, the anionic OA ligands are replaced with short ones such as acetate ions through solid‐state ligand exchange using methyl acetate (MeOAc)‐based ligand solution. The desired film thickness of CsPbI_3_ PQD solids is achieved by repeating this process based on a layer‐by‐layer (LbL) assembly. Second, to replace residual cationic OLA ligands with short cationic ammonium ligands, the CsPbI_3_ PQD solids are post‐treated with ethyl acetate (EtOAc)‐based ligand solution.^[^ ^20^ ^]^ As such, the ligand exchange procedure using ionic short‐chain ligands dissolved in polar solvents enables the fabrication of thick and conductive CsPbI_3_ PQD solids as a photovoltaic absorber.^[^ ^21^, ^22^ ^]^ However, the ionic surface nature of PQDs induces the deterioration in optoelectrical properties and ambient stability of conductive PQD solids and restricts further improvement of photovoltaic performance in PQD solar cells.^[^ ^23^, ^24^ ^]^

During the ligand exchange, the polar solvents play a key role in dissolving ionic salts to supply short‐chain ligands while removing long‐chain ligands from the PQD surface.^[^ ^22^ ^]^ However, due to the ionic surface nature of PQDs, the polar solvents inevitably induce the removal of not only surface‐bound ligands, but also metal cations and halides from the PQD surface.^[^ ^25^, ^26^, ^27^, ^28^ ^]^ The removal of such PQD surface components (i.e., surface‐bound ligands, metal cations, and halides) leads to the generation of surface traps on the PQDs, such as uncoordinated Pb^2+^ sites, which can act as non‐radiative recombination centers and serve as penetration pathways for destructive species such as oxygen and water molecules.^[^ ^29^, ^30^ ^]^ Moreover, it is difficult to efficiently passivate these surface traps using conventional ionic short‐chain ligands because of their labile and weak chemical bonds onto the ionic PQD surface; thus, limiting the improvement of optoelectrical properties and ambient film stability of CsPbI_3_ PQD photovoltaic absorber.^[^ ^31^, ^32^ ^]^ Nevertheless, the conventional ligand exchange is inevitable for fabricating thick and conductive CsPbI_3_ PQD photovoltaic absorbers. Therefore, after conventional ligand exchange, PQD surface should be stabilized through efficient surface trap passivation to improve the photovoltaic performance and stability of PQD solar cells.

Herein, we demonstrate a facile surface stabilization strategy of ligand‐exchanged PQD solids using a covalent short‐chain ligand, which can be dissolved in nonpolar solvents and strongly bound to the PQD surface via Lewis‐base interactions, to simultaneously improve the photovoltaic performance and stability of CsPbI_3_ PQD solar cells. The surface stabilization strategy using covalent short‐chain ligands for the CsPbI_3_ PQD photovoltaic absorbers fabricated using the conventional ligand exchange procedure should satisfy several considerations as follows: 1) the ligand should have a stronger binding affinity with uncoordinated Pb^2+^ sites than any other adsorbed destructive species,^[^ ^33^, ^34^ ^]^ 2) the ligand should have a short chain that does not hinder the inter‐dot charge transport within the PQD thin‐films, and 3) PQDs with an ionic nature have a sensitive surface to polar solvents.^[^ ^35^, ^36^, ^37^ ^]^ Thus, the solvent dissolving the covalent short‐chain ligands should be carefully selected to suppress the additional loss of the PQD surface components and further generation of surface traps.

Considering these points, a triphenylphosphine oxide (TPPO) short‐chain ligand, which can be dissolved in nonpolar solvents (i.e., octane) and covalently bound to the uncoordinated Pb^2+^ sites on the PQD surface via Lewis‐base interactions, is employed in this study. Through in‐depth analysis of the PQD surface, we confirm that the uncoordinated Pb^2+^ sites generated after the conventional ligand exchange procedure could be strongly passivated without any further loss of PQD surface components using covalent short‐chain TPPO ligand dissolved in a nonpolar solvent octane. Therefore, after treatment with the TPPO ligand solution dissolved in octane, the CsPbI_3_ PQD photovoltaic absorbers show improved PL intensity and ambient film stability owing to the significantly reduced surface trap density. Consequently, the CsPbI_3_ PQD solar cells after treatment with the TPPO ligand solution dissolved in octane exhibit enhanced photovoltaic performance up to a *PCE* of 15.4% and maintained more than 90% of its initial efficiency after storage under ambient conditions for 18 days.

## 2 Result and Discussion

### 2.1 Conventional Ligand Exchange Procedure of CsPbI_3_ PQDs

**Figure 1:**
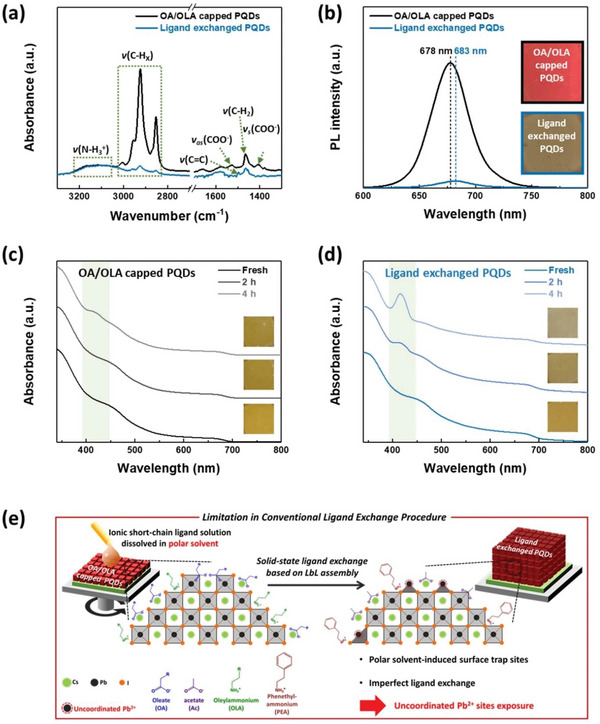
a) FT‐IR and b) PL spectra of the OA/OLA‐capped and ligand‐exchanged PQD solids, respectively. The insets in b exhibit photographs of each PQD solid under UV irradiation. UV–vis absorbance spectra and photographs of c) OA/OLA‐capped and d) ligand‐exchanged PQD solids before and after storing under harsh relative humidity of 50–60% RH condition. e) Schematic illustration of conventional ligand exchange procedure.

Monodispersed OA/OLA‐capped CsPbI_3_ PQDs with a crystal size of ≈10 nm were synthesized via a hot‐injection method (Figure S1, Supporting Information). To fabricate ligand‐exchanged PQD solids for solar cell applications, surface‐bound OA and OLA ligands were exchanged with anionic acetate using sodium acetate (NaOAc) solution dissolved in MeOAc and short‐chain cationic ammonium using phenethylammonium iodide (PEAI) solution dissolved in EtOAc, respectively, via a two‐step ligand exchange procedure (See Experimental Section). Fourier‐transform infrared (FT‐IR) spectra of the OA/OLA‐capped and ligand‐exchanged PQD solids were measured to investigate the relative amount of surface‐bound ligands on each PQD solid before and after the ligand exchange (**Figure** **1**a). Compared to the OA/OLA‐capped PQD solids, the ligand‐exchanged PQD solids showed decreased IR peak intensities of the oleyl (*v*(C—H_X_) and *v*(C—H_2_)) and carboxylate groups (*v*_as_(COO^−^) and *v*_s_(COO^−^)). This indicates that the OA and OLA ligands were removed from the PQD surface, and the OA ligands were replaced with acetate ions. Meanwhile, although the OLA ligands were removed from the ligand‐exchanged PQDs, the IR peak intensity of ammonium (*v*(N—H_3_^+^)) was maintained and the aromatic double bonding peak (*v*(C=C)) increased, suggesting that the PEA cations were incorporated into the OLA ligand‐removed sites.^[^ ^20^ ^]^ Therefore, it can be concluded that the long alkyl chain ligands were removed from the PQD surface, and the ionic short‐chain ligands were anchored onto the PQD surface after ligand exchange.

The optical characteristics of CsPbI_3_ PQD solids fabricated based on ligand exchange were also identified using PL spectroscopy measurements (Figure 1b). The PL emission peak of ligand‐exchanged PQD solids (683 nm) was red‐shifted compared to that of OA/OLA‐capped PQD solids (678 nm), indicating a reduced inter‐dot distance of the neighboring PQDs after ligand exchange.^[^ ^38^ ^]^ Simultaneously, a significantly decreased PL intensity was observed in the ligand‐exchanged PQD solids (Figure 1b and inset photograph images). The decreased PL intensity can be explained by two causes as follows; 1) increased energy transfer phenomena between PQDs, resulting from the reduced inter‐dot distance after ligand exchange,^[^ ^39^ ^]^ 2) increased non‐radiative recombination at the ligand‐exchanged PQD solids, resulting from the generation of surface traps by incomplete ligand exchange.^[^ ^23^, ^24^ ^]^ The second case suggests that the surface traps on the PQDs could act as a non‐radiative recombination center and reduce the PL intensity. In other words, it can be explained that the number of surface traps on the PQDs was increased by ligand exchange procedure leading to significantly decreasing the PL intensity.

CsPbI_3_ PQDs undergo phase transformation from the cubic phase to the orthorhombic phase, when destructive species such as moisture and oxygen molecules penetrate the PQD surface through numerous surface trap sites and accelerate the degradation process.^[^ ^40^, ^41^, ^42^ ^]^ Therefore, the generation of surface traps on PQDs after ligand exchange can be qualitatively demonstrated through the investigation of ambient stability of PQD solids. The ambient stability tests of OA/OLA‐capped and ligand‐exchanged PQD solids were performed using UV–vis spectroscopy by stored under harsh relative humidity (RH) condition of 50–60%, respectively. After 4 h, the OA/OLA‐capped PQD solids maintained their pristine UV–vis absorbance and brownish film color (Figure 1c). However, the UV–vis absorbance spectra of the ligand‐exchanged PQD solids exhibited an additional absorbance peak at ≈430 nm and bleaching of the brownish film color (Figure 1d). These results were attributed to the phase transformation of cubic phase CsPbI_3_ perovskite to the orthorhombic phase. We also confirmed by Tauc plot that the absorbance peak at ≈430 nm corresponds to orthorhombic phase of CsPbI_3_ perovskite with an optical bandgap of 2.83 eV (Figure S2, Supporting Information).^[^ ^43^, ^44^ ^]^ This phenomenon demonstrates that ligand‐exchanged PQD solids suffer from phase degradation of the cubic phase to the orthorhombic phase, indicating that the surface trap sites of the PQD solids which are generated after the ligand exchange procedure accelerated the penetration of the destructive species to the PQD surface.^[^ ^12^, ^21^, ^45^ ^]^ Consequently, we confirmed by PL spectra and ambient film stability that conventional ligand exchange using ionic short‐chain ligands imperfectly exchanged the OA and OLA ligands due to their labile and weak bonding with the PQD surface, resulting in the generation of unexpected surface trap sites in PQD solids.

Figure 1e illustrates the limitations of the conventional ligand exchange procedure using polar solvent‐based ionic short‐chain ligand solution as explained above. To fabricate conductive CsPbI_3_ PQD solids for thick photovoltaic absorbers, it is essential that the native long‐chain OA and OLA ligands are removed through LbL assembly‐based solid‐state ligand exchange using polar solvent‐based ligand solution. In this process, because of the ionic nature of the PQDs, polar solvents inevitably induce the loss of the PQD surface components and expose the uncoordinated Pb^2+^ sites, which are the major surface traps on the PQDs.^[^ ^21^, ^46^ ^]^ Moreover, these uncoordinated Pb^2+^ sites are not sufficiently passivated by ionic short‐chain ligands because of imperfect ligand exchange. Consequently, the ligand‐exchanged CsPbI_3_ PQD solids suffer from unfavorable charge transport and degradation of film stability. These results imply that the surface traps such as uncoordinated Pb^2+^ sites of the ligand‐exchanged PQD solids should be diminished without the loss of PQD surface components, through a further PQD surface stabilization strategy using a tailored ligand solution.

### 2.2 Solvent Effects on Ligand‐Exchanged CsPbI_3_ PQD Solids

**Figure 2:**
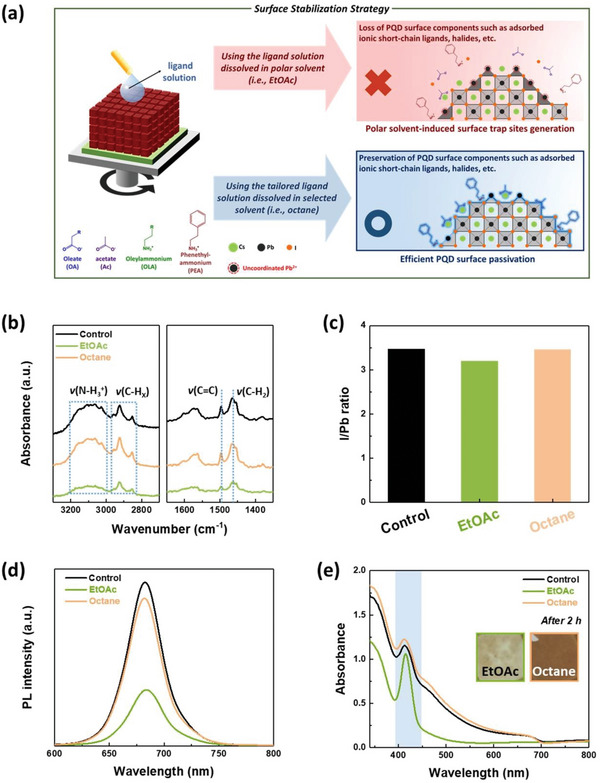
a) Schematic illustration showing the importance of the solvents used in the surface passivation process for efficient surface stabilization strategy. b) FT‐IR spectra of control, EtOAc‐, and octane‐treated CsPbI_3_ PQD solids, respectively. c) I/Pb ratio extracted from the XPS spectra and d) PL spectra of each CsPbI_3_ PQD solid. e) UV–vis absorbance spectra of each CsPbI_3_ PQD solid stored under 50–60% RH condition for 2 hours. The inset photographs show the film stability of EtOAc‐ and octane‐treated CsPbI_3_ PQD solids, respectively.

For efficient surface stabilization of the ligand‐exchanged CsPbI_3_ PQD solids, a tailored ligand solution should be employed, in which new short‐chain ligands can be strongly bound to the uncoordinated Pb^2+^ sites without the loss of PQD surface components. During this surface stabilization process, the solvent containing the new short‐chain ligands may negatively affect the surface state of the PQDs, similar to the polar solvents adopted in conventional ligand exchange, as explained in Figure 1. Therefore, the solvent used in this process should be carefully selected to suppress both the loss of the PQD surface components, including the adsorbed ionic short‐chain ligands, and further generation of surface trap sites (**Figure** **2**a).

To determine the appropriate solvent for the surface stabilization, the control CsPbI_3_ PQD solids, which were fabricated via a conventional ligand exchange procedure, were rinsed with neat solvents to investigate the solvent effect on the PQD surface. Among the various solvents that were widely adopted in the ligand exchange of PQDs, we selected the representative polar solvent EtOAc and the nonpolar solvent octane.^[^ ^22^, ^47^, ^48^ ^]^ The FT‐IR spectra showed a clear difference in the relative amounts of surface‐bound ligands on the PQDs after the respective EtOAc and octane treatments (Figure 2b). The control CsPbI_3_ PQD solids exhibited the IR peaks related to alkyl, ammonium, and aromatic double bonding groups, originated from the surface‐bound ligands which were remained after ligand exchange. Compared to the control CsPbI_3_ PQD solids, the FT‐IR spectra of the EtOAc‐treated CsPbI_3_ PQD solids exhibited decreased IR peaks related to alkyl, ammonium, and aromatic double bonding groups. However, the octane‐treated CsPbI_3_ PQD solids maintained the IR peaks of the surface‐bound ligands. This indicates that EtOAc results in the unwanted removal of surface‐bound ligands, whereas octane completely preserves them.

To observe the elemental compositions of the CsPbI_3_ PQD solids treated with different neat solvents, the atomic ratio of the CsPbI_3_ PQD solids was obtained through X‐ray photoelectron spectroscopy (XPS) measurements (Table S1, Supporting Information). Compared with the control CsPbI_3_ PQD solids, the atomic C/Pb and N/Pb ratios were maintained after octane treatment, whereas these values were reduced after EtOAc treatment (Figure S3, Supporting Information). This result reveals that EtOAc treatment led to the loss of surface‐bound ligands on the PQDs. The O 1s core‐level spectra of the CsPbI_3_ PQD solids treated with different solvents also support the observed solvent effects on the loss of surface‐bound ligands. The O 1s core‐level spectra were deconvoluted into two sub‐peaks at 532.5 and 531.2 eV which were attributed to the carboxylate and lead hydroxide (Pb‐OH), respectively (Figure S4, Supporting Information).^[^ ^18^ ^]^ Compared to the octane‐treated CsPbI_3_ PQD solids, the EtOAc‐treated CsPbI_3_ PQD solids showed higher Pb—OH/COO^−^ ratio indicating the surface hydroxylation due to the loss of surface‐bound ligands from the PQD surface after EtOAc treatment. Furthermore, the I/Pb ratio of the CsPbI_3_ PQD solids provides the direct evidence for losing the iodides from the PQD surface after neat EtOAc treatment because the CsPbI_3_ PQDs have the Cs‐ and I‐terminated (100) facets (Figure 2c).^[^ ^49^ ^]^ The octane‐treated CsPbI_3_ PQD solids showed an I/Pb ratio consistent with that of the control CsPbI_3_ PQD solids. However, the I/Pb ratio decreased after EtOAc treatment, indicating that iodides were lost from the PQD surface. The loss of surface‐bound ligands and iodides from the PQD surface by EtOAc treatment can lead to the further generation of surface trap sites within the CsPbI_3_ PQD solids.

We measured the PL spectra of the ligand‐exchanged CsPbI_3_ PQD solids treated with different neat solvents to investigate the solvent effects on the PQDs in detail (Figure 2d). The PL intensity of the CsPbI_3_ PQD solids treated with EtOAc was significantly reduced, indicating increased non‐radiative recombination due to the generation of numerous surface trap sites within the CsPbI_3_ PQD solids. This generation of surface trap sites can be attributed to the loss of surface‐bound ligands and iodides caused by the EtOAc treatment. In contrast, the octane‐treated CsPbI_3_ PQD solids showed a slightly reduced PL intensity compared to the control CsPbI_3_ PQD solids. Although octane treatment can almost preserve the surface‐bound ligands and ions of PQDs, the dynamic PQD surface can induce continuous ionic ligand adsorption and desorption. Therefore, the slight decrease in the PL intensity after octane treatment might be attributed to the dynamic and labile bonding between the surface ionic ligands and the PQDs.^[^ ^31^ ^]^ In addition, we measured the UV–vis absorbance changes before and after different neat solvent treatments (Figure 2e; Figure S5, Supporting Information). The EtOAc‐treated CsPbI_3_ PQD solids exhibited the occurrence of an additional absorbance peak at ≈430 nm after 2 h stored under 50–60% RH condition, indicating the severe phase degradation from the cubic phase to the orthorhombic phase. In contrast, the UV–vis absorbance spectrum of the octane‐treated CsPbI_3_ PQD solids showed a similar trend to that of the control CsPbI_3_ PQD solids. Therefore, it can be concluded that the octane treatment had no significant effect on the PQD surface states.

We also employed other conventional polar solvents (2‐pentanol and MeOAc) and nonpolar solvents (toluene and hexane), which are widely used in the research field of PQD‐based optoelectronic devices, to investigate the solvent effects on the ligand‐exchanged CsPbI_3_ PQD solids. The FT‐IR spectra revealed that the polar solvents much more significantly induced the removal of surface‐bound ligands than the nonpolar solvents, although the degree of ligand removal might be different according to the polarity and dielectric constant of the polar solvents (Figure S6, Supporting Information).^[^ ^22^ ^]^ Consequently, the polar solvent‐treated CsPbI_3_ PQD solids suffer from larger non‐radiative recombination and lower ambient film stability compared to the nonpolar solvent‐treated PQD solids (Figures S7 and S8, Supporting Information). Therefore, it can be concluded that nonpolar solvents are suitable for the surface stabilization strategy of the ligand‐exchanged CsPbI_3_ PQD solids. Among the nonpolar solvents, the octane‐treated CsPbI_3_ PQD solids showed the most preserved PL intensity, UV–vis absorbance, and ambient film stability compared with the control CsPbI_3_ PQD solids.

### 2.3 Surface Stabilization Strategy Using Covalent TPPO Ligand Dissolved in Octane

**Figure 3:**
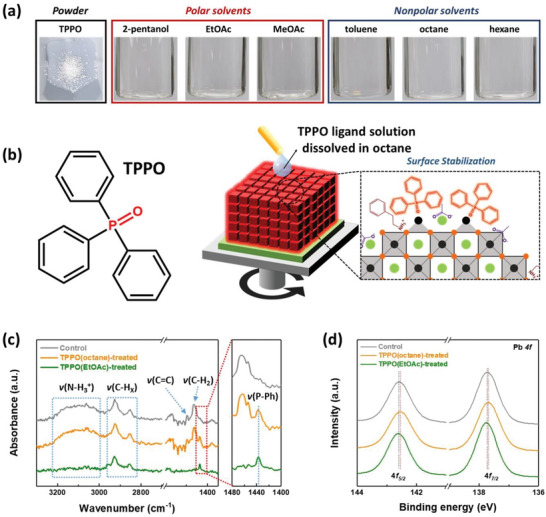
a) The photographs of TPPO powder and the solubility of TPPO in various polar and nonpolar solvents. b) Chemical structure of TPPO and schematic illustration of surface stabilization strategy using TPPO ligand solution dissolved in octane. c) FT‐IR spectra of TPPO molecule, control, TPPO(octane)‐, and TPPO(EtOAc)‐treated CsPbI_3_ PQD solids, respectively. d) Pb 4f spectra extracted from the XPS spectra of each CsPbI_3_ PQD solid.

Covalent ligands that have lone pair electrons can be strongly coordinated with electron‐deficient uncoordinated Pb^2+^ sites through Lewis‐base interactions.^[^ ^33^, ^46^, ^50^ ^]^ Therefore, tailored covalent short‐chain ligands dissolved in nonpolar solvents (i.e., octane) are suitable for the surface stabilization of the ligand‐exchanged PQD solids. Among the various covalent short‐chain ligands, TPPO can be considered as an appropriate candidate for the surface stabilization of PQDs because of its strong binding affinity with Pb^2+^, which is confirmed by previous studies.^[^ ^33^, ^51^ ^]^ Moreover, unlike conventional ionic short‐chain ligand salts that are not thoroughly dissolved in nonpolar solvents, the TPPO ligand powder can be well dissolved in nonpolar solvents at high concentrations (0.5 mg mL^−1^; **Figure** **3**a; Figure S9, Supporting Information). Therefore, a TPPO ligand solution dissolved in octane (hereafter, TPPO(octane)) was selected to realize the surface stabilization of the ligand‐exchanged CsPbI_3_ PQD solids. Figure 3b illustrates the chemical structure of TPPO and facile surface stabilization strategy via TPPO(octane) treatment of the CsPbI_3_ PQD solids. We expected that the strong covalent short‐chain TPPO ligand could effectively passivate the uncoordinated Pb^2+^ sites through Lewis‐base interactions and suppress the loss of PQD surface components by using the nonpolar solvent octane as a ligand‐dissolving solvent.

FT‐IR measurements were performed to explore the effects of the TPPO ligand solution, which are dissolved in the respective EtOAc and octane, on the surface‐bound ligands of PQDs (Figure 3c). The IR peak intensities of the alkyl, ammonium, and aromatic double bonding groups were maintained after TPPO(octane) treatment, indicating that the ionic surface‐bound ligands were preserved because octane can suppress the loss of PQD surface components. In addition, an IR peak at the wavenumber of ≈1439 cm^−1^, originating from the stretching vibration of the P‐phenyl (*v*(P—Ph)) bonding of TPPO, was clearly observed.^[^ ^52^ ^]^ This provides obvious evidence of the incorporation of TPPO ligand on the PQD surface. After the TPPO ligand solution dissolved in EtOAc (hereafter, TPPO(EtOAc)) treatment, the IR peak of P‐phenyl bonding was also observed; however, the representative IR peaks of the alkyl, ammonium, and aromatic double bonding groups significantly decreased because of the loss of surface‐bound ligands by polar EtOAc solvent effect. Furthermore, the TPPO(octane)‐treated PQD solids showed the IR peak of P=O stretching vibration (*v*(P=O)) at the wavenumber of ≈1173 cm^−1^, which is lower than that of the molecular TPPO (1183 cm^−1^) (Figure S10, Supporting Information). This indicates that the P=O bonding of TPPO weakens because of the interaction between uncoordinated Pb^2+^ sites on the PQD surface and TPPO ligand.^[^ ^33^ ^]^

The XPS spectra provide clear evidence of the changes in the PQD surface composition after the TPPO(octane) and TPPO(EtOAc) treatments (Figure 3d). Compared to the control CsPbI_3_ PQD solids, the Pb 4f peaks shifted to lower binding energy region after TPPO(octane) treatment, suggesting that the electron‐deficient Pb^2+^ was occupied by the lone pair electron of TPPO through Lewis‐base interactions.^[^ ^24^, ^53^ ^]^ For the same reason, the possibility of surface Cs^+^ passivation after TPPO(octane) treatment is described (Figure S11, Supporting Information). However, the binding energy shifts in Pb 4f and Cs 3d did not occur after TPPO(EtOAc) treatment, indicating insufficient passivation of uncoordinated Pb^2+^ sites on the PQD surface. This phenomenon can be explained by the loss of PQD surface components induced by polar EtOAc solvent, which results in the generation of additional uncoordinated Pb^2+^ sites on the PQD surface. This was also confirmed by the Pb 4f peaks of neat EtOAc‐treated PQD solids, which exhibit a shift to a higher binding energy compared to that of the control‐ and TPPO(EtOAc)‐treated PQD solids (Figure S12, Supporting Information). This phenomenon suggests that the neat EtOAc treatment results in the generation of the electron‐deficient uncoordinated Pb^2+^ sites that have higher binding energy between nucleus and electrons compared to atomic Pb. Although the TPPO ligand can bind to uncoordinated Pb^2+^ sites after TPPO(EtOAc) treatment, it does not prevent the loss of surface components caused by the EtOAc polar solvent. Therefore, it can be concluded that TPPO(octane) treatment is much more appropriate for the surface stabilization of the ligand‐exchanged CsPbI_3_ PQD solids compared to TPPO(EtOAc).

### 2.4 Optoelectrical Properties and Ambient Stability of Surface‐Stabilized PQD Solids

**Figure 4:**
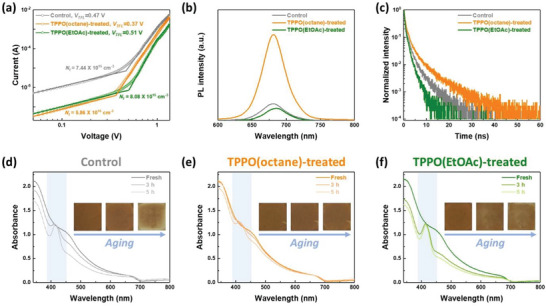
a) Trap density (N_t_) calculated from the V_TFL_ using the SCLC curves of electron‐only devices composed of control, TPPO(octane)‐, and TPPO(EtOAc)‐treated CsPbI_3_ PQD solids, respectively. b) PL and c) TRPL decay spectra of each CsPbI_3_ PQD solid. UV–vis absorbance spectra and film photographs of d) control, e) TPPO(octane)‐treated, and f) TPPO(EtOAc)‐treated CsPbI_3_ PQD solids stored under 50–60% RH condition, respectively.

We fabricated the electron‐only devices composed of the ligand‐exchanged CsPbI_3_ PQD solids before and after surface stabilization and then performed the space‐charge‐limit current (SCLC) measurements to estimate their trap density (N_t_). The N_t_ values of the CsPbI_3_ PQD solids were calculated using the trap‐filled limit voltage (V_TFL_) obtained from the SCLC curves of each electron‐only device (**Figure** **4**a). The TPPO(octane)‐treated device showed lower V_TFL_ and N_t_ values (0.37 V and 5.86 × 10^15^ cm^−3^, respectively) than those of the control device (V_TFL_ = 0.47 V and N_t_ = 7.44 × 10^15^ cm^−3^, respectively). This indicates that the TPPO(octane) treatment can efficiently diminish the surface trap sites, including uncoordinated Pb^2+^ sites, and suppress solvent‐induced additional side effects. However, the TPPO(EtOAc)‐treated device exhibited higher V_TFL_ and N_t_ values (V_TFL_ = 0.51 V and N_t_ = 8.08 × 10^15^ cm^−3^, respectively) than those of the control device. This phenomenon suggests that TPPO(EtOAc) treatment induces the further generation of surface trap sites due to the polar nature of the EtOAc solvent. The resultant tendency of N_t_ values observed in CsPbI_3_ PQD solids before and after surface stabilization is also consistent with that of their PL spectra measurements as shown in Figure 4b. Compared to the control CsPbI_3_ PQD solids, the TPPO(octane)‐treated CsPbI_3_ PQD solids exhibited a higher PL intensity. This can be attributed to the reduction of surface traps in the PQD solids due to the TPPO(octane) treatment, which significantly suppressed non‐radiative recombination. However, the TPPO(EtOAc)‐treated CsPbI_3_ PQD solids showed a lower PL intensity, which might be attributed to the increased surface traps (Figure 4a). Time‐resolved PL (TRPL) spectroscopy was performed to further investigate the charge carrier dynamics in the PQD solids (Figure 4c; Table S2, Supporting Information). The TPPO(octane)‐treated CsPbI_3_ PQD solids exhibited an average carrier lifetime of a 2.70 ns, which is longer than that of the control and TPPO(EtOAc)‐treated CsPbI_3_ PQD solids (1.35 and 0.99 ns, respectively). This result indicates that the non‐radiative recombination was considerably suppressed after TPPO(octane) treatment, consistent with the observations in the SCLC and PL spectra results.

We also explored the ambient film stability of each CsPbI_3_ PQD solid stored under 50–60% RH condition using UV–vis absorbance measurements to confirm the effect of surface stabilization strategy on the ligand‐exchanged PQD solids. After 5 h, reduction in absorbance and the appearance of the orthorhombic phase absorbance peak at ≈430 nm was identified in both the control and TPPO(EtOAc)‐treated CsPbI_3_ PQD solids. In contrast, the TPPO(octane)‐treated CsPbI_3_ PQD solids retained almost their initial absorbance. In addition, while both the control and TPPO(EtOAc)‐treated CsPbI_3_ PQD solids lost their initial brownish film color, the TPPO(octane)‐treated CsPbI_3_ PQD solids maintained their film color, indicating their robustness against harsh humidity conditions (Figure 4d–f and inset photographs). The robustness of the TPPO(octane)‐treated CsPbI_3_ PQD solids was also confirmed under 20–30% RH condition. After 60 days, the TPPO(octane)‐treated CsPbI_3_ PQD solids maintained their initial UV–vis absorbance and film color, whereas the control CsPbI_3_ PQD solids exhibited an orthorhombic absorbance peak and film color bleaching (Figure S13, Supporting Information). An X‐ray diffraction (XRD) pattern was employed to evaluate the differences in the structural stability of the CsPbI_3_ PQD solids (Figure S14, Supporting Information). Initially, the XRD spectra of both control and TPPO(octane)‐treated CsPbI_3_ PQD solids exhibited (100), (110), and (200) plane peaks, which correspond to the black cubic phase of CsPbI_3_ PQDs. After 60 days, new plane peaks corresponding to the photo‐inactive and yellow orthorhombic phase were observed in the control CsPbI_3_ PQD solids. However, the cubic phase plane peaks were thoroughly preserved in the TPPO(octane)‐treated CsPbI_3_ PQD solids, which is in good agreement with the corresponding UV–vis spectra and aging film images.

### 2.5 Photovoltaic Performance and Stability of Surface‐Stabilized CsPbI_3_ PQD Solar Cells

**Figure 5:**
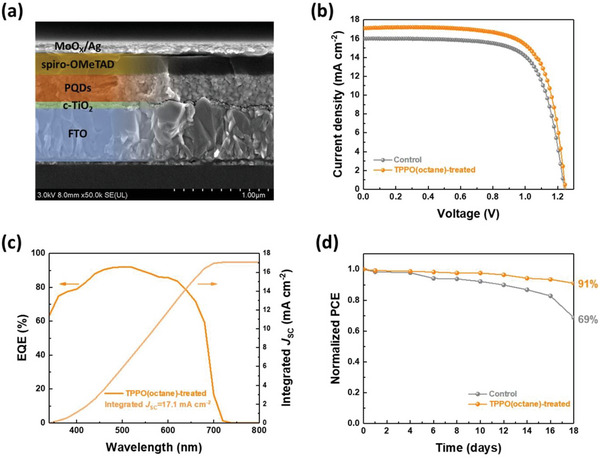
a) Cross‐sectional SEM image of the TPPO(octane)‐treated CsPbI_3_ PQD solar cell. b) *J–V* curves of control and TPPO(octane)‐treated CsPbI_3_ PQD solar cells, respectively. c) EQE spectra with integrated *J*_SC_ value of TPPO(octane)‐treated CsPbI_3_ PQD solar cells. d) Long‐term device stability of each CsPbI_3_ PQD solar cell, stored under ambient condition with 20–30% RH for 18 days.

To investigate the impact of TPPO(octane) treatment on the photovoltaic performance and ambient stability of solar cells, we fabricated solar cells using both control and TPPO(octane)‐treated CsPbI_3_ PQD solids. The cross‐sectional scanning electron microscopy (SEM) image described the device structure of the CsPbI_3_ PQD solar cells (**Figure** **5**a). The current density–voltage (*J*–*V*) curves of the CsPbI_3_ PQD solar cells, obtained under AM 1.5G illumination exhibited an improved photovoltaic performance of the TPPO(octane)‐treated CsPbI_3_ PQD solar cells compared to the control CsPbI_3_ PQD solar cells (Figure 5b and **Table** **1**). The TPPO(octane)‐treated CsPbI_3_ PQD solar cells exhibited a *PCE* of 15.4% with a short‐circuit current density (*J*_SC_) of 17.1 mA cm^−2^ which is markedly higher than that of control CsPbI_3_ PQD solar cells (*PCE* of 14.1% with a *J*_SC_ of 16.0 mA cm^−2^). Moreover, the integrated *J*_SC_ value, calculated from the external quantum efficiency (EQE) spectra of TPPO(octane)‐treated CsPbI_3_ PQD solar cells reached up to a 17.1 mA cm^−2^ which is good agreement with a *J*_SC_ value, identified from the *J*–*V* measurement (Figure 5c). In addition, the statistical tendency of the photovoltaic performance of the control and TPPO(octane)‐treated CsPbI_3_ PQD solar cells was also consistent with the obtained values of the *J–V* measurements (Figure S15, Supporting Information; Table 1).

**Table 1:** Photovoltaic performance parameters and average values of each of 21 control and TPPO(octane)‐treated CsPbI_3_ PQD solar cells

		*V*_OC_ [V]	*J*_SC_ [mA cm^−2^]	*FF* [%]	*PCE* [%]
Control	Champion Average	1.24 1.24 ± 0.02	16.0 15.5 ± 0.6	71.2 70.5 ± 1.4	14.1 13.6 ± 0.5
TPPO(octane)‐treated	Champion Average	1.25 1.25 ± 0.01	17.1 16.8 ± 0.3	71.9 70.3 ± 1.8	15.4 14.7 ± 0.7

FF: Fill factor.

To further understand the improved photovoltaic performance of TPPO(octane)‐treated CsPbI_3_ PQD solar cells, we measured *J*_SC_ and open‐circuit voltage (*V*_OC_) of the solar cells as a function of the incident light intensity from 1 to 100 mW cm^−2^ (Figure S16, Supporting Information). Both the control and TPPO(octane)‐treated CsPbI_3_ PQD solar cells exhibited exponential factors (*α*) close to unity in the logarithmic plots of the light‐intensity‐dependent *J*_SC_, indicating that both CsPbI_3_ PQD solar cells almost suppressed bimolecular recombination under short‐circuit conditions.^[^ ^13^ ^]^ Meanwhile, light‐intensity‐dependent *V*_OC_ measurements were conducted to investigate the trap‐assisted carrier recombination of both the control and TPPO(octane)‐treated CsPbI_3_ PQD solar cells. The TPPO(octane)‐treated CsPbI_3_ PQD solar cells exhibited a linear slope of 1.28 kT q^−1^ (k: Boltzmann constant, T: Kelvin temperature, and q: elementary charge), which was lower than that of the control CsPbI_3_ PQD solar cells (1.48 kT q^−1^), suggesting that TPPO(octane) treatment can effectively prevent trap‐assisted carrier recombination.^[^ ^23^ ^]^ Therefore, the improved photovoltaic performance of TPPO(octane)‐treated CsPbI_3_ PQD solar cells can be attributed to the reduced surface trap density of the PQD solids after TPPO(octane) treatment. Finally, we performed a long‐term device stability test, stored under 20–30% RH without encapsulation (Figure 5d). After 18 days of storage, the TPPO(octane)‐treated CsPbI_3_ PQD solar cells exhibited over 90% retention of their initial *PCE* value, while the control CsPbI_3_ PQD solar cells degraded rapidly. The improved device stability of the TPPO(octane)‐treated CsPbI_3_ PQD solar cells was ascribed to the sufficient passivation of uncoordinated Pb^2+^ sites without any additional loss of PQD surface components after TPPO(octane) treatment.

## 3 Conclusion

In this study, a facile CsPbI_3_ PQD surface stabilization strategy was demonstrated to improve the optoelectrical properties and ambient stability of CsPbI_3_ PQD photovoltaic absorbers for efficient and stable solar cells. The covalent short‐chain TPPO ligand can efficiently passivate the uncoordinated Pb^2+^ sites which are generated after the conventional ligand exchange procedure because of their strong Lewis‐base interactions. In addition, the TPPO ligand can be dissolved in a nonpolar solvent octane, thereby suppressing the further loss of PQD surface components, such as surface‐bound ligands and iodides, and the generation of new surface trap sites. Owing to the synergetic effects of the tailored TPPO ligand and the stable octane solvent, the TPPO(octane)‐treated CsPbI_3_ PQD photovoltaic absorber exhibited improved optoelectrical properties and ambient film stability. Consequently, the solar cells with the TPPO(octane)‐treated CsPbI_3_ PQD photovoltaic absorber achieved an enhanced *PCE* value of 15.4% with more improved device stability than that of control solar cells. This study provides new insights into the surface passivation strategies of PQDs for high‐performance solar cells by introducing a facile surface stabilization strategy with a covalent short‐chain ligand and nonpolar solvent, which differs from conventional ligand passivation methods.

## 4 Experimental Section

### Chemicals

Lead iodide (II) (PbI_2_, 99.999%), 1‐octadecene (technical grade 90%), oleic acid (technical grade 90%), cesium carbonate (Cs_2_CO_3_, 99.99%), *n*‐hexane (anhydrous), *n*‐octane (99%), toluene (anhydrous), 2‐pentanol (2‐PeOH, 99%), lithium bis(trifluoromethylsulfonyl)imide (Li‐TFSI, 98%), and 2‐*n*‐pentylpyridine were purchased from Alfa Aesar. Oleylamine (technical grade 70%), sodium acetate (NaOAc, 99.995%), chlorobenzene (anhydrous 99.8%), and acetonitrile (99.8%), triphenylphosphine oxide (TPPO, 98%), were purchased from Sigma–Aldrich. Phenethylammonium iodide (PEAI) was purchased from GreatcellSolar. 2,20,7,70‐Tetrakis(*N*,*N*‐di‐*p*‐methoxyphenylamine)‐9,90‐spirobifluorene (Spiro‐OMeTAD, ≥ 99.5%) was purchased from Lumtec. Methyl acetate (MeOAc, ≥ 99.5%) and ethyl acetate (EtOAc, ≥ 99.5%) were purchased from Duksan. TiO_2_ precursor solution (0.15 M), and TiCl_4_ aqueous solution (2 M) were purchased from Sharechem. Phenyl‐C61‐butyric acid methyl ester (PCBM, ≥99.95%) was purchased from 1‐Materials.

### Synthesis of CsPbI_3_ PQDs

For the synthesis of Cs‐oleate solution, 0.407 g of Cs_2_CO_3_, 1.25 mL of oleic acid, and 20 mL of 1‐octadecene were put into a 250 mL of three‐necked flask and degassed at 120 °C. After the Cs_2_CO_3_ was completely dissolved, the Cs‐oleate solution was stored in Ar‐filled atmosphere at 90 °C. For the synthesis of CsPbI_3_ PQDs, 0.5 g of PbI_2_ and 25 mL of 1‐octadecene were put into a 100 mL of three‐necked flask. After the flask was degassed for 30 min at 115 °C, 2.5 mL of oleic acid and 2.5 mL of oleylamine were added into the flask and degassed until the PbI_2_ was fully dissolved. After the temperature was increased to 185 °C, filling with Ar gas, the 2 mL of Cs‐oleate precursor was injected rapidly into the flask. After 5 s, the reaction was quenched into the ice bath.

### Purification of CsPbI_3_ PQDs

The 30 mL of crude solution was mixed with the 60 mL of MeOAc and the mixture was centrifuged at 5000 rpm for 3 min. The precipitates were dispersed in the 10 mL of *n*‐hexane and the 14 mL of MeOAc was added. The mixture was centrifuged at 5000 rpm for 3 min. The precipitates were re‐dispersed in the 30 mL of *n*‐hexane and the solution was centrifuged at 5000 rpm for 3 min. The CsPbI_3_ PQDs dispersed in hexane were stored in the dark at 4 °C. After 24 h, hexane was dried completely under vacuum. Finally, CsPbI_3_ PQDs pellets were re‐dispersed in octane with a concentration of 75 mg mL^−1^.

### Fabrication of CsPbI_3_ PQD Solar Cells

Pre‐patterned fluorine‐doped tin oxide (FTO) substrates were cleaned by sonicating using detergent, deionized water, acetone, and isopropyl alcohol. After that, the FTO substrates were treated by UV/O_3_ for 20 min. To fabricate an electron‐transporting layer, the c‐TiO_2_ precursor solution was spin‐coated on the FTO substrates for 30 s at 3000 rpm, and the substrates were annealed at 500 °C for 1 h. After cooled to room temperature, the substrates were immersed in a 120 mm TiCl_4_ aqueous solution at 70 °C for 1 h. After that, the substrates were rinsed with deionized water and annealed at 500 °C for 1 h. To fabricate ligand‐exchanged CsPbI_3_ PQD solids act as a photovoltaic absorber, the CsPbI_3_ PQDs solution was spin‐coated onto the FTO/c‐TiO_2_ for 20 s at 1000 rpm and for 5 s at 2000 rpm. The CsPbI_3_ PQD solids were treated with 1 mg mL^−1^ of NaOAc solution dissolved in MeOAc for 3 s and washed using neat MeOAc. This procedure was repeated 4–6 times for desired film thickness. After that, the CsPbI_3_ PQD solids was treated with 1 mg mL^−1^ of PEAI solution dissolved in EtOAc for 10 s and then washed using neat MeOAc. For surface stabilization, the ligand‐exchanged CsPbI_3_ PQD solids were additionally treated with 0.5 mg mL^−1^ of TPPO solution and then washed using neat solvent. To fabricate a hole‐transporting layer, the spiro‐OMeTAD solution prepared by mixing 72.3 mg of spiro‐OMeTAD, 1 mL of chlorobenzene, 29 µL of 2‐*n*‐pentylpyridine, and 17.5 µL of Li‐TFSI solution in acetonitrile with a concentration 520 mg mL^−1^. The prepared spiro‐OMeTAD solution was spin‐coated on the CsPbI_3_ PQDs photovoltaic absorber for 30 s at 4000 rpm. Finally, 15 nm of MoO_X_ and 120 nm of Ag electrodes were deposited with a thermal evaporator. For space‐charge‐limited current (SCLC) measurement, an electron‐only device of FTO/TiO_2_/CsPbI_3_ PQDs/PCBM/Au structure was fabricated. PCBM dissolved in chlorobenzene with the concentration of 20 mg mL^−1^ was spin‐coated at 3000 rpm for 30 s under a N_2_‐filled glove box, and the Au electrode was deposited with a thermal evaporator.

### Characterization

Absorption spectra were measured using an Jasco V‐770 UV–vis–NIR spectrophotometer. High‐resolution transmission electron microscopy (HR‐TEM) images were obtained using a Hitachi HF‐3300 and W electron source. Photoluminescence (PL) spectra were measured by utilizing a Horiba Scientific Flouromax‐4 spectrophotometer. Time‐resolved PL (TRPL) decay curves were measured by utilizing a Hamamatsu Quantaurus‐Tau C11367 spectrometer. The PL lifetimes were estimated by fitting the TRPL decay containing the fluorescence decay (*τ*_1_), the intermediate fluorescence decay (*τ*_2_), and similarly ultralong‐lived fluorescence decay (*τ*_3_) following the triexponential equation:^[^ ^20^ ^]^

(1) $$y\;\left(t\right)=y_{0}\;+\;A_{1}\exp\left(-\frac{t}{\tau_{1}}\right)+\;A_{2}\exp\left(-\frac{t}{\tau_{2}}\right)+\;A_{3}\exp\left(-\frac{t}{\tau_{3}}\right)$$

where *A*_1_, *A*_2_, and *A*_3_ are the relative ratio of amplitude in each component of decay curves.

Fourier‐transform infrared (FT‐IR) spectroscopy were obtained by utilizing a Thermo Scientific Nicolet6700. X‐ray photoelectron spectroscopy (XPS) were measured by using utilizing a Thermo Scientific ESCALAB 250Xi analyzer. X‐ray diffraction (XRD) data were obtained by utilizing a Panalytical Empyrean X‐ray diffractometer. Cross‐sectional scanning electron microscopy (SEM) image was obtained by using a Hitachi SU8230 equipment. The current density–voltage (*J–V*) characteristics were measured by utilizing a Newport Oriel Sol 3A solar simulator with a Keithley 2400 sourcemeter under air mass 1.5 (AM 1.5G) and 100 mW cm^−2^ (1 sun) illumination. The *J–V* curves of the solar cells were obtained with reverse scan direction from 1.3 to −0.05 V with active area of 0.075 cm^2^. The SCLC of electron‐only devices was obtained from the *J‒V* measurements using a Keithley 2400 sourcemeter under dark conditions. Trap‐state density (N_t_) as calculated from trap‐filled limit voltage (V_TFL_), obtained from the SCLC curve, using the equation as follows.

(2) $$V_{\mathrm{TFL}}=\frac{qN_{t}L^{2}}{2\varepsilon \varepsilon_{0}}\;$$

where *q* is the elementary charge, *L* is the thickness of CsPbI_3_ PQD solids, *ε* is the relative permittivity of CsPbI_3_ (*ε* = 6.32*ε*_0_) and *ε*_0_ is the vacuum permittivity, respectively.

## Conflict of Interest

The authors declare no conflict of interest.

## Supporting information

Supporting Information

## Data Availability

The data that support the findings of this study are available from the corresponding author upon reasonable request.
