# Supplementary material for: Stabilized Perovskite Quantum Dot Solids via Nonpolar Solvent Dispersible Covalent Ligands
Source: Adv Sci (Weinh). 2023 Jun 4;10(23):2301793. doi: 10.1002/advs.202301793 (PMC10427392; doi:10.1002/advs.202301793)
Supplement: Supplementary file 1 — Supporting Information [file ADVS-10-2301793-s001.pdf]

ADVANCED SCIENCE
Open Access

Supporting Information

for *Adv. Sci.*, DOI 10.1002/advs.202301793

Stabilized Perovskite Quantum Dot Solids via Nonpolar Solvent Dispersible Covalent Ligands

Sanghun Han, Gayoung Seo, Taeyeong Yong, Seongmin Choi, Younghoon Kim and Jongmin Choi**

Supporting Information

Stabilized Perovskite Quantum Dot Solids via Nonpolar Solvent Dispersible Covalent Ligands

Sanghun Han‡, Gayoung Seo‡, Taeyeong Yong, Seongmin Choi, Younghoon Kim, Jongmin Choi**

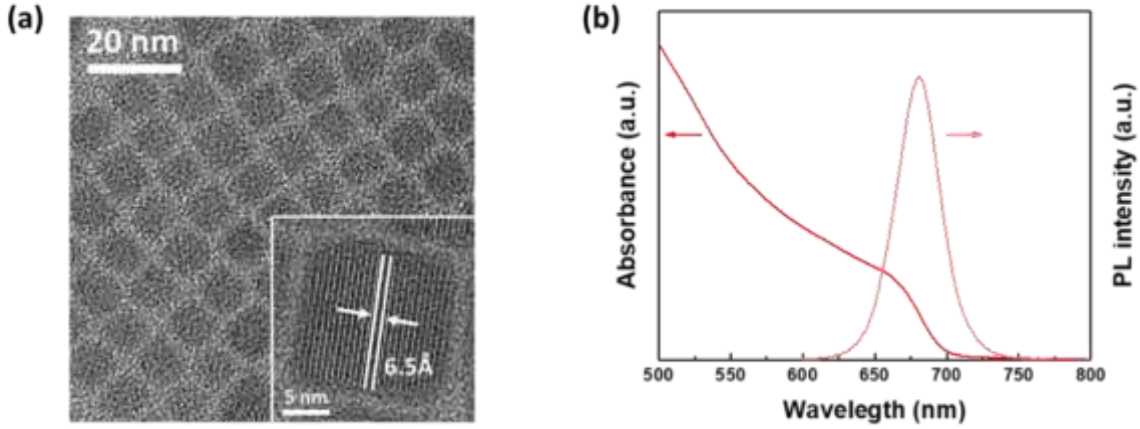

Figure S1. (a) HR-TEM image of $CsPbI_3$ PQDs. The inset image indicates lattice fringe of cubic α-phase $CsPbI_3$ PQDs. (b) Solution-phase UV-Vis absorbance and PL spectra of $CsPbI_3$ PQDs.

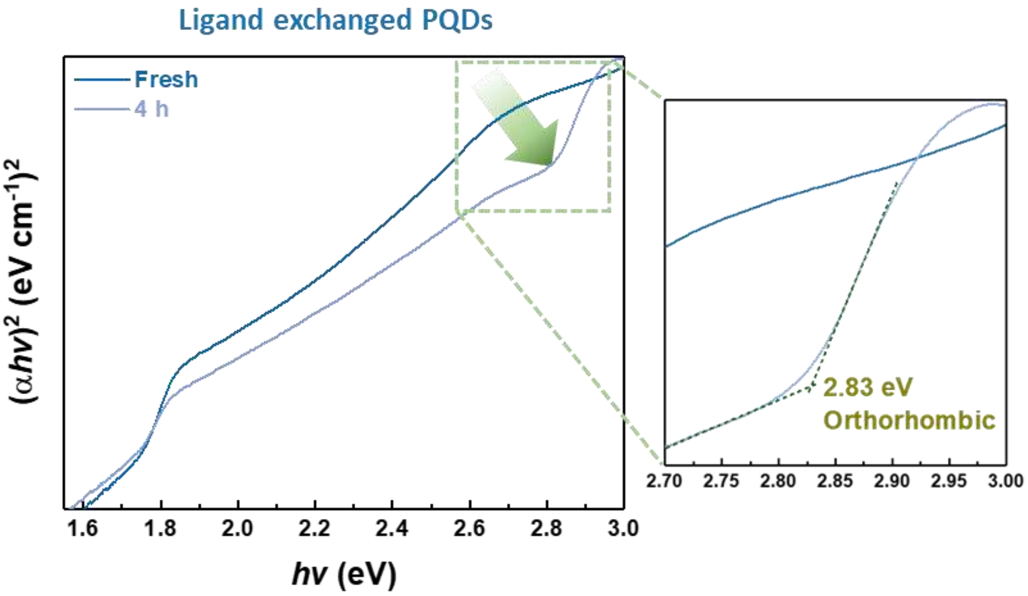

Figure S2. Tauc plot and optical band gap, originated from the UV-Vis spectra of ligand-exchanged PQD solids.

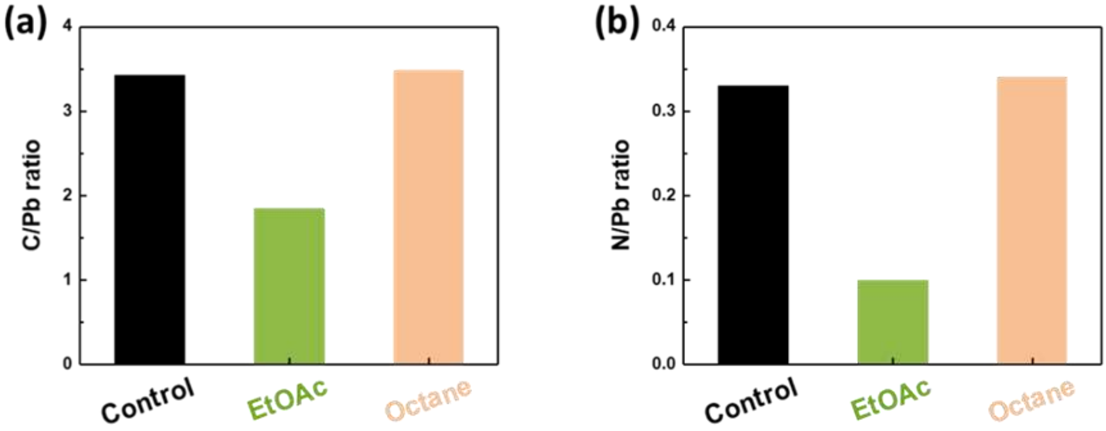

Figure S3. (a) C/Pb and (b) N/Pb atomic ratios extracted from XPS measurement of control, EtOAc-, and octane-treated $CsPbI_3$ PQD solids.

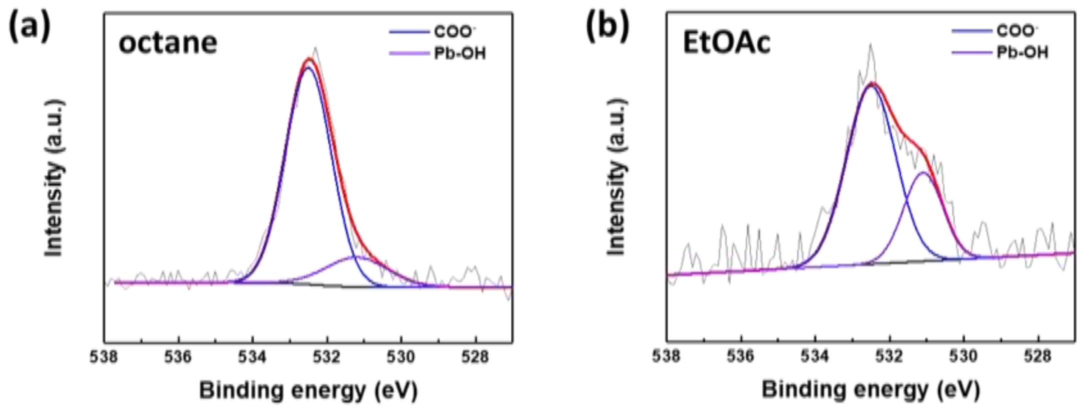

Figure S4. Deconvoluted XPS O 1s core level spectra of (a) octane- and (b) EtOAc-treated $CsPbI_3$ PQD solids.

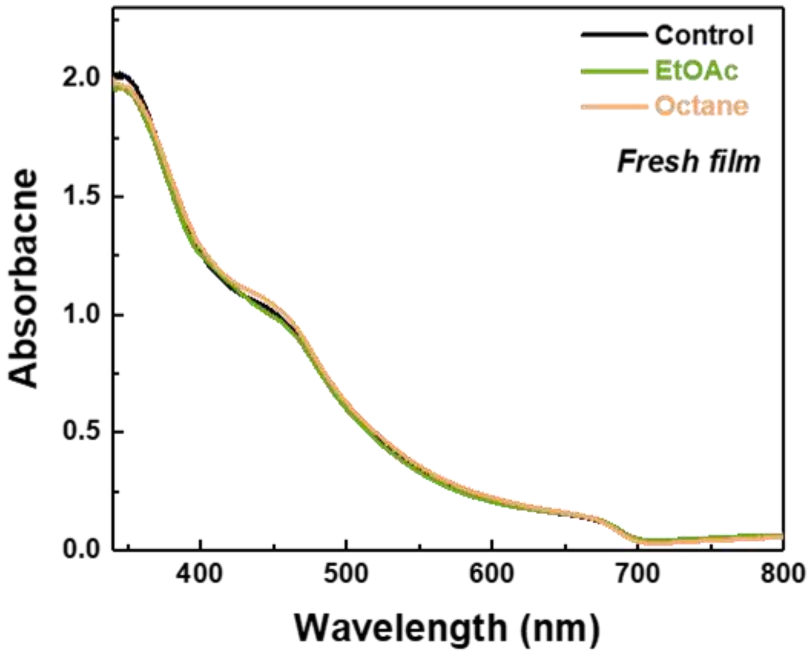

Figure S5. UV-Vis absorbance spectra of control, EtOAc-, and octane-treated $CsPbI_3$ PQD solids in their initial state, measured under 50-60% RH condition.

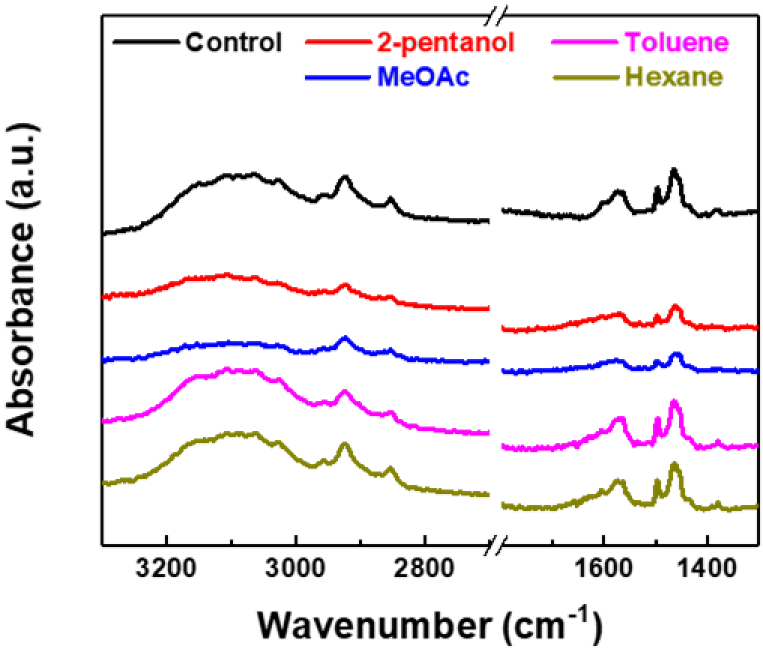

Figure S6. FT-IR spectra of the ligand-exchanged $CsPbI_3$ PQD solids before and after treated with various neat polar solvents (2-pentanol and MeOAc) and nonpolar solvents (toluene and hexane).

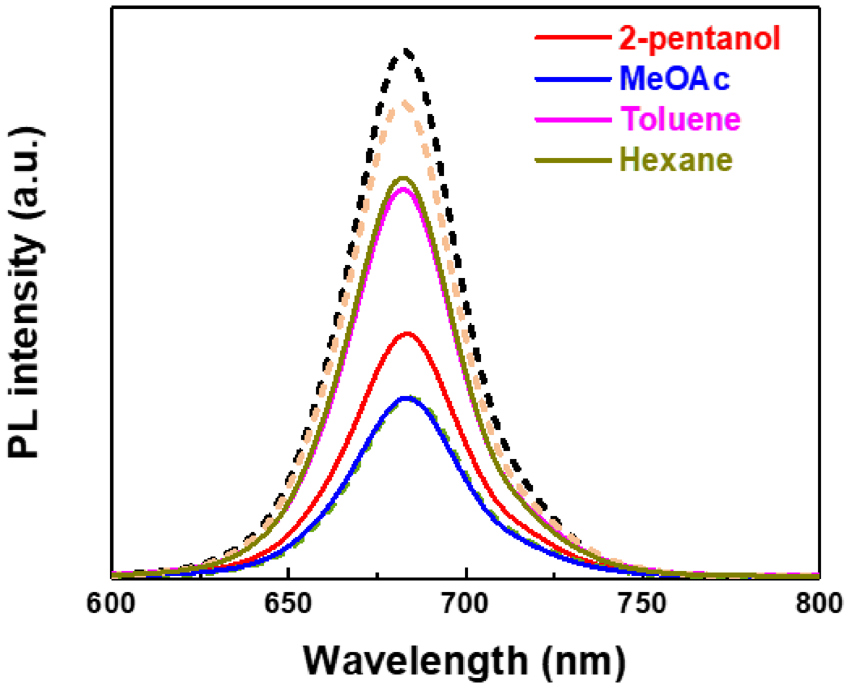

Figure S7. PL spectra of the ligand-exchanged $CsPbI_3$ PQD solids after treated with various neat polar solvents (2-pentanol and MeOAc) and nonpolar solvents (toluene and hexane). The black, orange, and green dash-line indicate PL spectra of control, octane-, and EtOAc-treated $CsPbI_3$ PQD solids, respectively, which are described in **Figure 2d**.

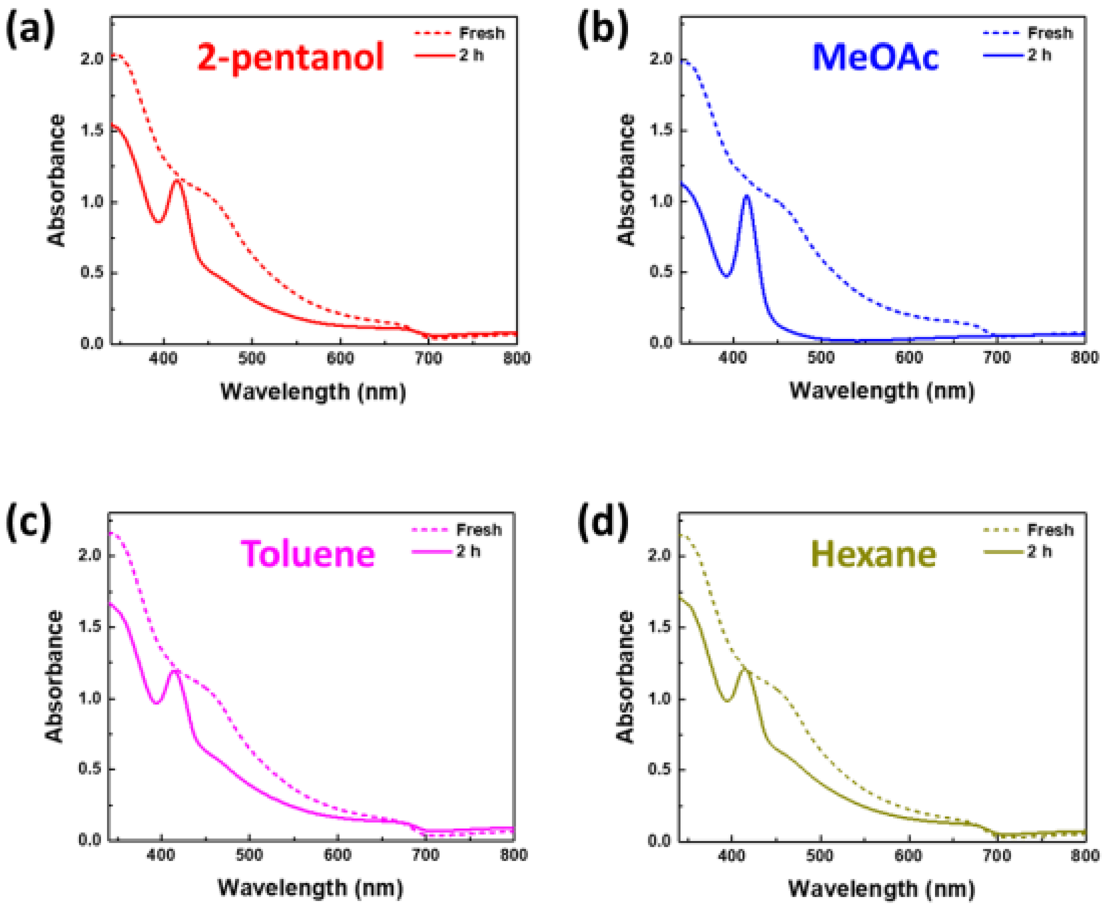

Figure S8. UV-Vis absorbance spectra of the ligand-exchanged $CsPbI_3$ PQD solids treated with (a) 2-pentanol, (b) MeOAc, (c) toluene, and (d) hexane, before and after stored under 50-60% RH condition for 2 hours.

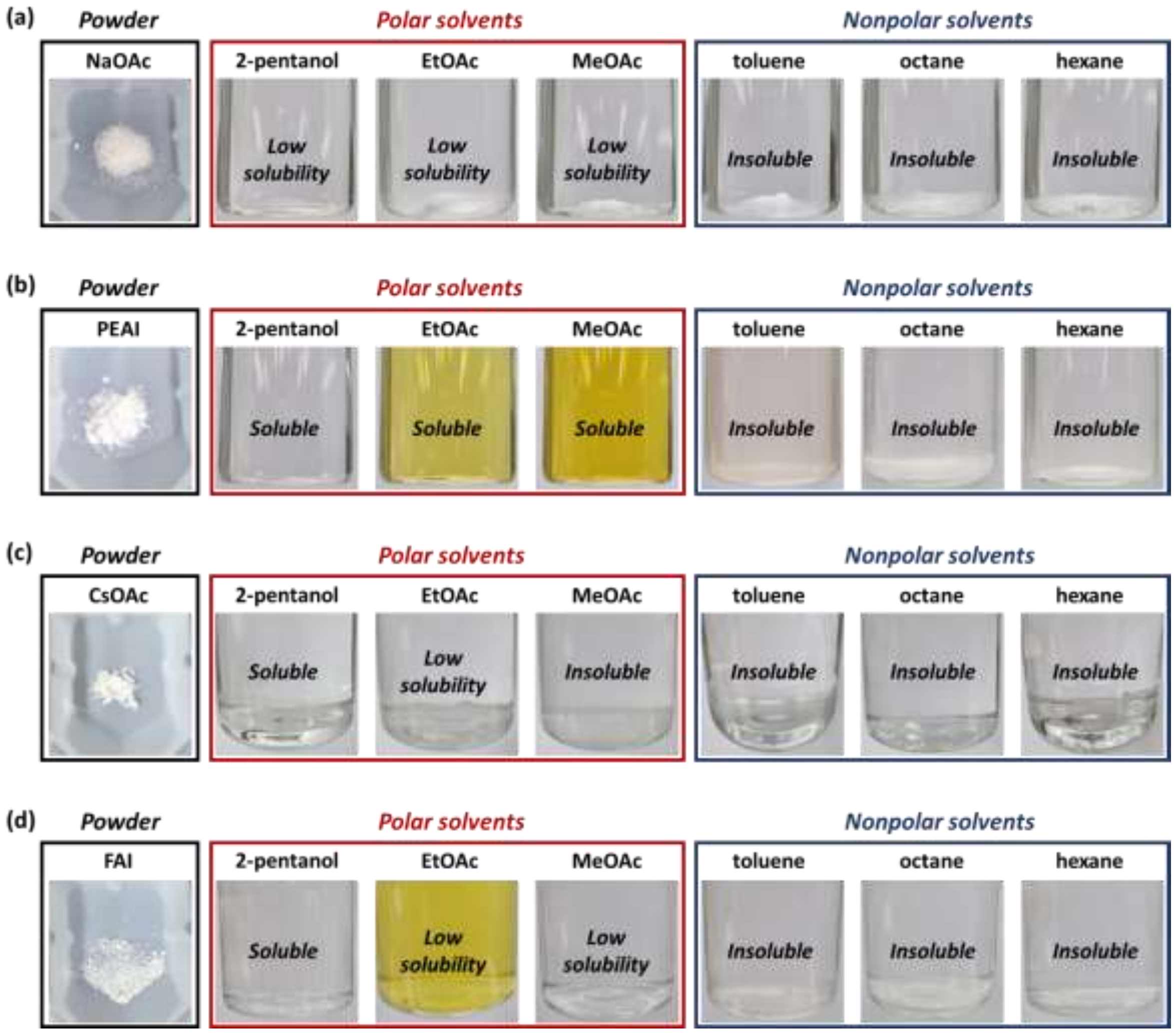

Figure S9. The photographs of (a) NaOAc, (b) PEAI, (c) cesium acetate (CsOAc), and (d) formamidinium iodide (FAI) powders and the solubility of respective ionic ligand salts in various solvents according to the solvent polarity.

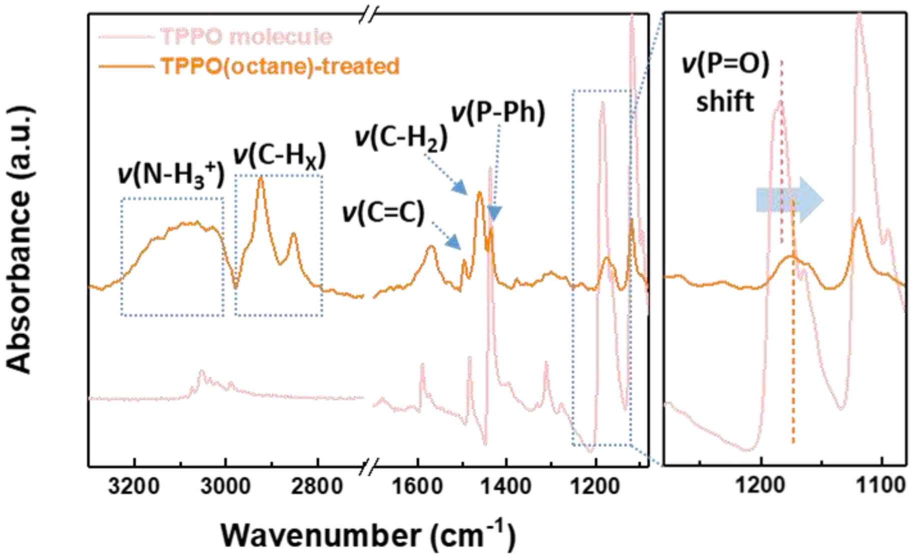

Figure S10. Peak shift of (P=O) stretching vibration of TPPO(octane)-treated PQD solids compared to TPPO molecule.

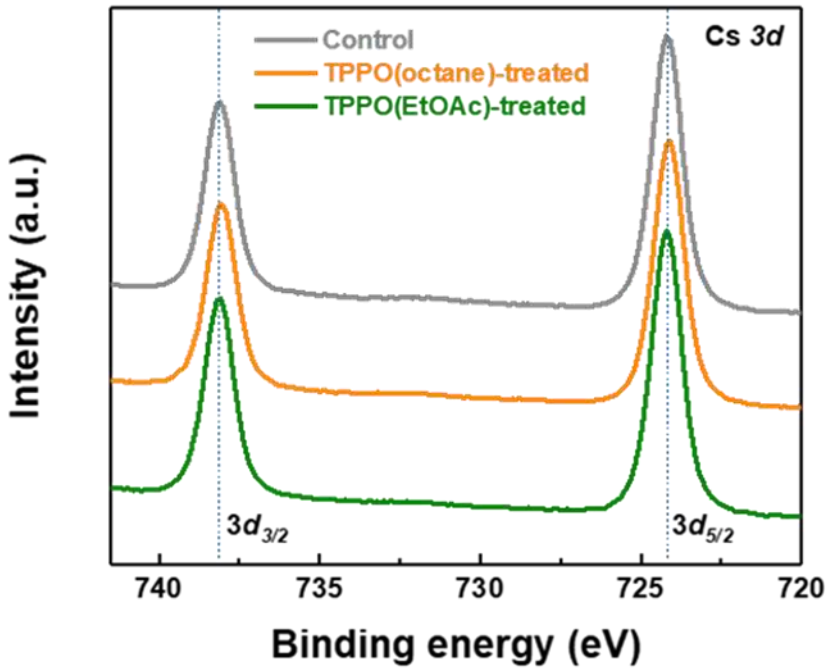

Figure S11. Cs 3d spectra extracted from the XPS measurement of control, TPPO(octane)-, and TPPO(EtOAc)-treated $CsPbI_3$ PQD solids.

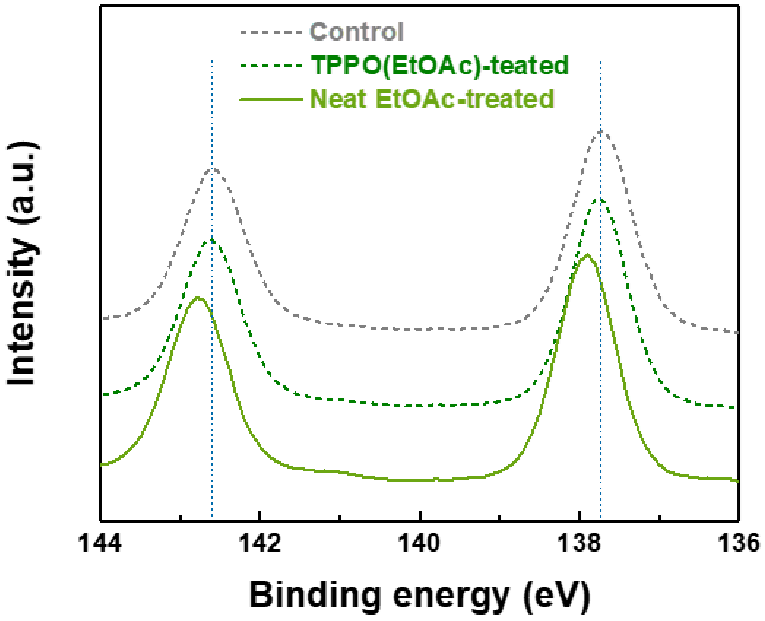

Figure S12. Pb 4f spectra extracted from the XPS spectra of control, TPPO(EtOAc)-, and EtOAc-treated PQD solids. The dash-line of control and TPPO(EtOAc)-treated PQD solids are described in **Figure 3d**.

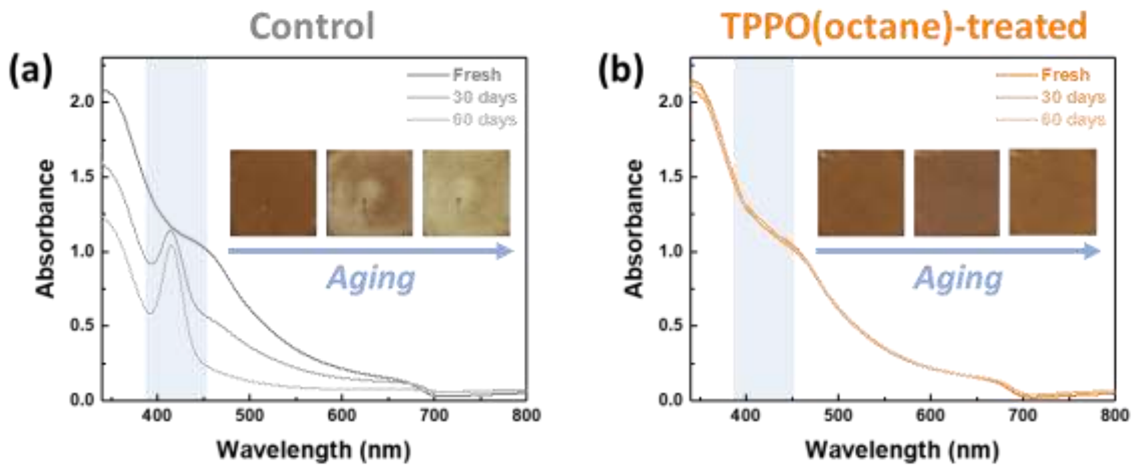

Figure S13. UV-Vis absorbance spectra and film photographs of (a) control and (b) TPPO(octane)-treated $CsPbI_3$ PQD solids stored under ambient condition with 20-30% RH.

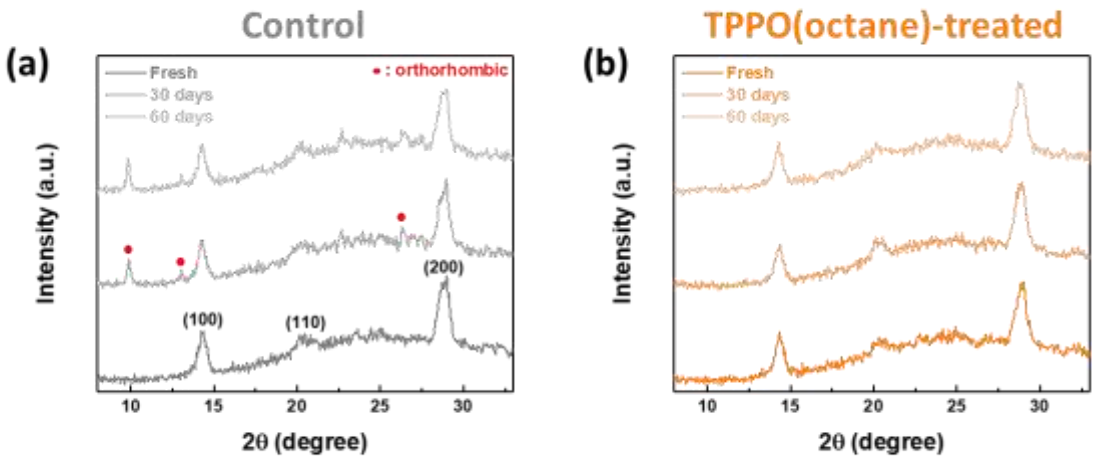

Figure S14. XRD patterns of (a) control and (b) TPPO(octane)-treated $CsPbI_3$ PQD solids stored under ambient condition with 20-30% RH.

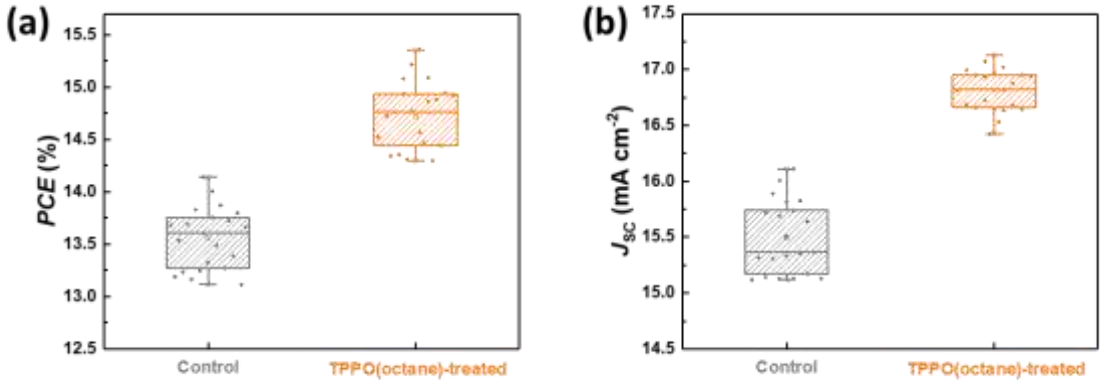

Figure S15. Device histograms of each of 21 control and TPPO(octane)-treated $CsPbI_3$ PQD solar cells, respectively: (a) *PCE* and (b) J_{SC}.

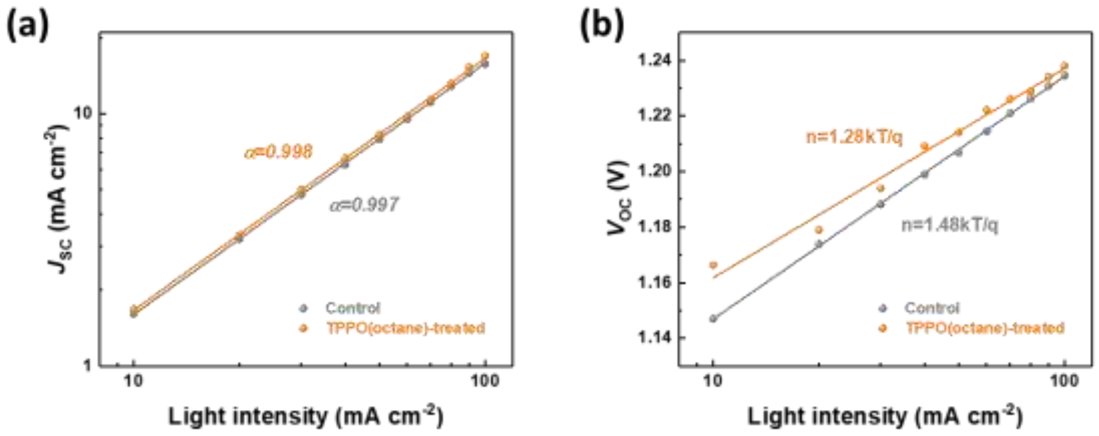

Figure S16. (a) Light-intensity-dependent J_{SC} and (b) V_{OC} of control and TPPO(octane)-treated $CsPbI_3$ PQD solar cell.

Table S1. Atomic ratio extracted from XPS measurement of control, octane-, EtOAc-, TPPO(octane)-, and TPPO(EtOAc)-treated $CsPbI_3$ PQD solids.

Name	C *1s*	O *1s*	N *1s*	Cs *3d*	Pb *4f*	I *3d*
Control	35.91	1.65	3.48	12.11	10.49	36.37
Octane-treated	34.87	5.61	3.38	11.57	10.02	34.55
EtOAc-treated	24.31	3.23	1.38	15.71	13.18	42.20
TPPO(octane)-treated	32.62	4.82	2.69	12.36	10.73	36.77
TPPO(EtOAc)-treated	34.60	4.72	2.15	12.38	10.79	35.36

Table S2. TRPL decay parameters of control, TPPO(octane)-, and TPPO(EtOAc)-treated $CsPbI_3$ PQD solids.

Name	A_1 (%)	T_1 (ns)	A_2 (%)	T_2 (ns)	A_3 (%)	T_3 (ns)	T_{ave} (ns)
Control	68.3	0.55	33.9	0.56	10.4	2.86	1.35
TPPO(octane)-treated	91.4	0.61	17.5	2.75	0.85	13.1	2.70
TPPO(EtOAc)-treated	61.3	0.52	41.2	0.52	7.83	2.40	0.99
